# Viral hepatitis in correctional facilities in the Northern Territory of Australia 2003–2017

**DOI:** 10.1186/s12879-021-06286-2

**Published:** 2021-06-16

**Authors:** Richard P. Sullivan, Rob Baird, Kevin Freeman, Hugh Heggie, Joshua S. Davis, Catherine S. Marshall, Jane Davies

**Affiliations:** 1 Charles Darwin University, Menzies School of Health Research, Casuarina, Northern Territory Australia; 2 Department of Infectious Diseases, Royal Darwin Hospital, Casuarina, Northern Territory Australia; 3 Territory Pathology, Royal Darwin Hospital, Casuarina, Northern Territory Australia; 4 St George & Sutherland Clinical School, UNSW, Kogarah, NSW Australia; 5 Northern Territory Department of Health, Northern Territory, Casuarina, Australia; 6 John Hunter Hospital, New Lambton Heights, New South Wales Australia

**Keywords:** Hepatitis B, Hepatitis C, Prisoner health, Northern Territory

## Abstract

**Background:**

The demographic of Northern Territory prison population differs than elsewhere in Australia and the prevalence of hepatitis B and hepatitis C may therefore be somewhat different from other jurisdictions. There has been no study which has specifically described the serological results of a large proportion of prisoners in Northern Territory correctional facilities over an extended period of time.

**Methods:**

This retrospective longitudinal study reviewed serological results and testing rates for hepatitis B, and hepatitis C performed in correctional facilities in the Northern Territory of Australia between July 1st, 2003 and June 30th, 2017.

**Results:**

The proportion of positive records over 14 years for hepatitis B surface antigen (HBsAg) was 641/12,066 (5.3, 95% CI 4.9–5.7), for hepatitis B core antibody (anti-HBc) 4937/12,138 (40.1, 95%CI 39.8–41.6), for hepatitis B surface antibody (anti-HBs) 6966/13,303 (52.4, 95% CI 51.5–53.2), and for hepatitis C antibody 569/12,153 (4.7, 95% CI 4.3–5.1). The proportion of prisoners tested for hepatitis B and hepatitis C has decreased since 2015, while a high proportion of prisoners remain non-immune to hepatitis B.

**Conclusion:**

There is a relatively high proportion of positive serological markers of hepatitis B, and a lower proportion of positive hepatitis C serology in the Northern Territory’s correctional facilities compared to overall Australian rates. As the proportion of prisoners tested for hepatitis B and C has decreased recently, and a high proportion of prisoners remain non-immune to hepatitis B, there are opportunities to increase testing and vaccination rates in this population.

## Background

Prison populations are at increased risk of exposure to viral hepatitis infection due to participation in a number of risk behaviours, such as injecting drug use, sharing tattooing and shaving equipment, fights, and sexual activity [1–6]. Chronic hepatitis B or hepatitis C infection can lead to liver cirrhosis and hepatocellular carcinoma, and are significant public health issues in Australia [7, 8]. Transmissible infectious diseases among prisoners may impact the health of the general community when prisoners are released back into the community, and hence the surveillance of these infections in correctional facilities is required [9].

There has been no study which has described the serological results of a large proportion of prisoners in Northern Territory correctional facilities. While the 2016 national prison entrants’ bloodborne virus survey for the Northern Territory showed hepatitis C serology was negative in all 39 participants, and hepatitis B surface antigen was positive in 2 of 38 (5%) [2], this represented only 1–2% of the over three thousand individuals received in Northern Territory correctional facilities that year [10]. Results from a larger proportion of prisoners tested would allow more precise estimate of the proportion who are serologically positive for viral hepatitis in these facilities.

The 2016 national prison entrants’ bloodborne virus survey found nationwide in a sample of 431 participants that the proportion of positive Hepatitis C serology was 22% while the proportion of positive anti-HBc, which denotes either current infection (HBsAg and anti-HBc positive) or past infection (HBsAg negative, anti-HBc positive) was 16% [2]. However, the demographic of Northern Territory prison population differs than elsewhere in Australia and the prevalence of hepatitis B and hepatitis C may therefore be somewhat different from other jurisdictions. 84% prisoners in the Northern Territory identify as Aboriginal or Torres Strait Islander, compared to a national average of 28% of the adult prisoner population [11]. A reported history of intravenous drug use in prisoners is also lower in the Northern Territory compared with the rest of Australia, while the Northern Territory has the highest imprisonment and recidivism rate of all states and territories [2, 10, 11].

The aims of this project were therefore to establish the proportion of positive results for serological markers of hepatitis B, and hepatitis C in all correctional facilities in the Northern Territory of Australia between 2003 and 2017 from a review of all serological results taken during this time and to review the rate of testing.

## Methods

We identified the results of serology for hepatitis B and hepatitis C of those who were incarcerated in correctional facilities of the Northern Territory of Australia between 2003 and 2017. There are two minimum to maximum correctional centres in the Northern Territory, which in 2017 had maximum capacities of 500 and 1048 respectively. There are males and females at both facilities and in 2017 less than 7% were incarcerated for illicit drug offences [10]. Testing for hepatitis B and C infection is typically offered at or just after reception, has been risk-based, and individuals are placed on care pathways with either general practitioner or specialist in-reach services. Outcomes for serology testing are reported as either positive, negative, equivocal or not tested, which is reported when the test is requested but it is not performed.

### Serological testing database

We identified records through Territory Pathology (Northern Territory public pathology laboratory), which covers all Northern Territory Government health care facilities including correctional facilities. All serological testing was performed at a single central lab located at Royal Darwin Hospital. Records of testing for hepatitis B and hepatitis C serology between 2003 and 2017 were generated by the pathology information system by searching for all results in which the location of testing was specified to a correctional facility in the Northern Territory. Serology for hepatitis B and hepatitis C was performed using Abbot AxSYM™ Microparticle Enzyme Immunoassay between 2003 and May 2007, and the Abbot Architect i2000SR-Chemiluminescent Microparticle Immunoassay between May 2007 and 2017. Hepatitis B and hepatitis C viral loads are performed in another state, and we did not have access to this data. Most serological testing was performed at another pathology provider (other than Territory Pathology) between 2007 and 2010 and we did not have access to the results from this provider.

### Database management

Names from the generated list of results from the pathology information system were converted to a 2 × 2 code (first 2 letters of first name followed by first two letters of last name), with all names removed from the dataset. The 2 × 2 code and date of birth was then converted to a unique study number. Data are currently stored on a password protected server at the Menzies School of Health Research, Darwin, and will be destroyed in five years.

We excluded indeterminate and equivocal results and combined the separate hepatitis B surface antigen (HBsAg), hepatitis B surface antibody (anti-HBs), and hepatitis B core antibody (anti-HBc) results of each individual in order to make a determination of the infectious and immune status of each individual. Results were determined to indicate infection (HBsAg positive), immunity by exposure (anti-HBc positive, anti-HBs positive, HBsAg negative), immunity by vaccination (anti-HBs positive, anti-HBc negative, HBsAg negative), isolated anti-HBc positive, or non-immunity (anti-HBs negative, anti-HBc negative, HBsAg negative). Anti-HBs was considered positive if titre was ≥10 mIU/mL.

### Data analysis

We analysed results for each financial year (July 1st to June 30th) and excluded any repeat tests which occurred on one individual within that year. For the hepatitis B serology, the first test of the year was included in the analysis. For the hepatitis C testing, if an individual tested negative and then had a positive result, the result for the year was taken as positive, while if an individual tested positive and then negative, they were excluded from the analysis for that year.

We calculated the rates of testing with numerator being the number of tests each financial year (July 1st to June 30th) and denominator being the number of unique individuals entering the correctional system in Northern Territory. The denominator is the actual throughput of prisoners each financial year and counts each individual only once [10]. This information was not available for July 1st 2009 to June 30th 2010, and July 1st 2010 to June 30th 2011 so the number of unique individuals entering the correctional system was estimated for these time periods as the average of the number of individuals entering from July 1st 2007 to June 30th 2008 and the number of individuals entering from July 1st 2011 to June 30th 2012. We calculated 95% confidence intervals using the exact binomial method. We did not stratify by Aboriginal and Torres Strait Islander status, as this was not available in the dataset.

We then calculated the total proportion of positive results for HBsAg, anti-HBc, anti-HBs, and hepatitis C serology over the 14-year study period by removing all duplicate test results so that each individual had one test only over the study period. Those who seroconverted (i.e. negative result followed by a positive result) during the study period were considered positive and their negative results were removed from the calculations. Those who hepatitis C serology was positive but on repeat testing were negative were excluded, while those who were ever HBsAg, anti-HBc, or anti-HBs were considered positive and were not excluded. We also expressed details on the age, and gender of those results which were positive for hepatitis C and HBsAg and performed logistic regression to determine if age or gender was associated with infection.

We determined the number of individuals who had more than one test for HBsAg and hepatitis C over the 14-year period and the proportion who seroconverted (negative to positive serology), and the median time to seroconversion. We also determined the number of individuals who had duplicate complete hepatitis B serology. The first and last hepatitis B serology tests were compared in this group.

The descriptive and statistical analyses described were performed using Stata statistical software (StataCorp. 2017. Stata Statistical Software: Release 15. College Station, TX: StataCorp LLC).

## Results

**Fig. 1:**
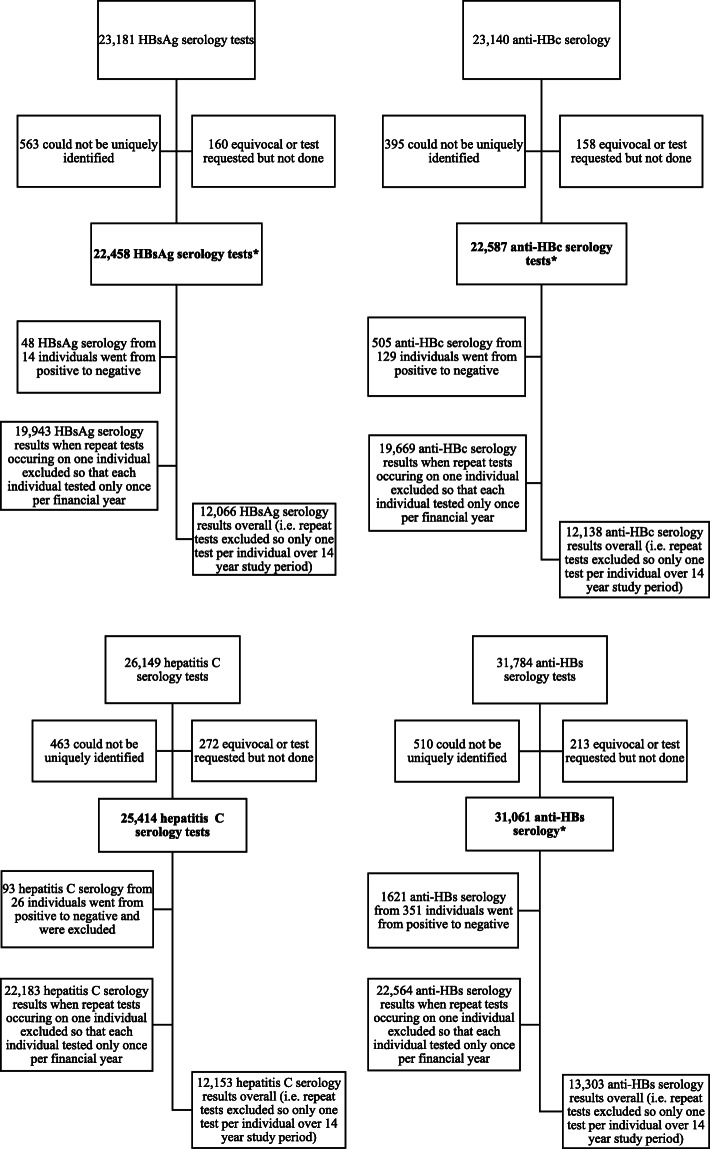
Total number for serology for HBsAg, anti-HBc, anti-HBs, and hepatitis C in NT correctional facilities 2003–2017. *22,397 were a part of a complete hepatitis B serology, which were used for the complete hepatitis B serology calculations. There were 19,930 unique complete hepatitis B serology tests when duplicates tests were removed within each year

**Fig. 2:**
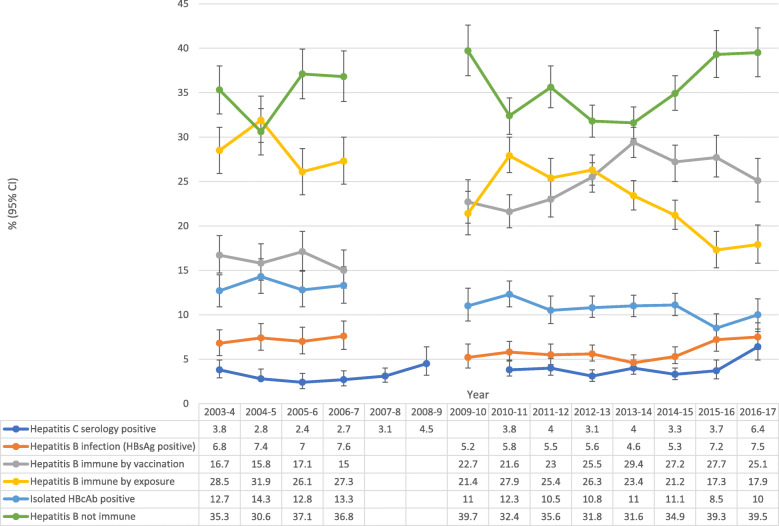
Positive serological markers of hepatitis B and C in correctional facilities of the Northern Territory 2003–2017. *Missing data between 2007 and 2010 as serological testing was performed at another pathology provider (other than Territory Pathology) and we did not have access to the results from this provider

There were 22,458 HBsAg, 22,587 anti-HBc, 31,061 anti-HBs, and 25,414 hepatitis C serology results available between the 1st July 2003 and the 30th June 2017. There were 22,397 complete hepatitis B serology tests, consisting of HBsAg, anti-HBs, and anti-HBc. Figure 1 shows the number of hepatitis B, and hepatitis C tests included and excluded in the study. There were significantly fewer tests between 2007 and 2010 as testing was performed at another pathology provider (other than Territory Pathology) during those years and we did not have access to these results. During these years there were multiple changes in testing policy and the ability to access to previous results, which resulted in fluctuations in rates of hepatitis B and C testing. Testing rates for hepatitis B and C dropped considerably from July 1st, 2015 (Table 1). The pathology provider has remained Territory Pathology from July 1st, 2015, and the reduced rate of testing reflects an actual decrease. The results of hepatitis B, and hepatitis C testing are given in Fig. 2.

**Table 1:** Testing rates for hepatitis B and hepatitis C in correctional facilities of the Northern Territory 2003–2017

Year	Number of distinct adults received	No. of unique^$^ hepatitis B tests	Prisoners tested hepatitis B (%)	No. of unique hepatitis C tests	Prisoners tested hepatitis C (%)
2003–4	1690	1208	71.5%	1455	86.1%
2004–5	1918	1256	65.5%	1435	74.8%
2005–6	1867	1147	61.4%	1497	80.2%
2006–7	2047	1106	54.0%	1519	74.2%
2007–8	2092	0	–	1708	81.6%
2008–9	2377	0	–	680	28.6%
2009–10	2526^¶^	1133	44.9%	26	1.0%
2010–11	2526^¶^	2027	80.2%	2046	81.0%
2011–12	2675	1640	61.3%	1643	61.4%
2012–13	3055	2702	88.4%	2696	88.2%
2013–14	3134	2710	86.5%	2537	81.0%
2014–15	3252	2413	74.2%	2759	79.3%
2015–16	3327	1351	40.6%	1253	37.7%
2016–17	3246	1237	38.1%	929	28.6%

^¶^estimate - information was not available for the 2009–2010, and 2010–2011 years so the number of unique individuals entering the correctional system was estimated as the average of the numbers entering in 2007–2008 and the numbers entering in 2011–2012

^$^ unique means only a single test per adult was counted per year

**Note:** Number of distinct adults received specifies the actual throughput of individual prisoners each year taken from publicly available information (i.e. individuals are only counted once even if there is recidivism) [10]

Removing all duplicate tests, the proportion of positive serological results over the 14 years for each of the virus markers were calculated. Records which demonstrated seroconversion (negative to positive serology) during the study period were considered positive. Records which went from a positive to a negative result for Hepatitis C were excluded and this occurred 26 times. Those who went from positive to negative for Hepatitis B serology were considered positive and occurred in 14 tested for HBsAg, 129 tested for anti-HBc, and 351 tested for anti-HBs. Over the 14 years, HBsAg was positive in 641 of 12,066 records (5.3, 95% CI 4.9–5.7), anti-HBc was positive in 4937 of 12,138 records (40.1, 95%CI 39.8–41.6), anti-HBs was positive in 6966 of 13,303 records (52.4, 95%CI 51.5–53.2), and hepatitis C serology was positive in 569 of 12,153 records (4.7, 95% CI 4.3–5.1). Results for HBsAg and hepatitis C serology by age and gender are given in Table 2.

**Table 2:** HBsAg and hepatitis C serology results by age and gender over the 14-year study period

	HBsAg (Positive Results/Number of Tests)	Hepatitis C (Positive Results/Number of Tests)
**Age < 21**	43/1896 (2.3, 95% CI 1.6–3.0)	15/1929 (0.8, 95% CI 0.4–1.3)
**Age 21–25**	83/2153 (3.9, 95% CI 3.1–4.8)	58/2242 (2.6, 95% CI 2.0–3.3)
**Age 26–30**	106/2114 (5.0, 95% CI 4.1–6.0)	113/2170 (5.2, 95% CI 4.3–6.2)
**Age 31–35**	114/1813 (6.3, 95% CI 5.2–7.5)	105/1855 (5.7, 95% CI 4.7–6.8)
**Age 36–40**	120/1563 (7.7, 95% CI 6.4–9.1)	94/1577 (6.0, 95% CI 4.8–7.2)
**Age 41–45**	73/1136 (6.4, 95% CI 5.1–8.0)	90/1083 (8.3, 95% CI 6.7–10.1)
**Age 46–50**	53/711 (7.5, 95% CI 5.6–9.6)	58/685 (8.5, 95% CI 6.5–10.8)
**Age 51–55**	30/367 (8.2, 95% CI 5.6–11.5)	23/358 (6.4, 95% CI 4.1–9.5)
**Age 56–60**	14/206 (6.8, 95% CI 3.8–11.1)	9/155 (5.5, 95% CI 2.5–10.1)
**Age > 61**	5/107 (4.7, 95% CI 1.5–10.6)	4/89 (4.5, 95% CI 1.2–11.1)
**Male**	601/10,586 (5.7, 95% CI 5.2–6.1)	499/10,725 (4.7, 95% CI 4.2–5.1)
**Female**	40/1480 (2.7, 95% CI 1.9–3.7)	70/1428 (4.9, 95% CI 3.8–6.2)

The median age for the positive hepatitis C serology results was 35 years (IQR 29–43) and for the negative hepatitis C serology results was 30 (IQR 23–38). The median age for the positive HBsAg results was 34 years (IQR 27–42) and for the negative HBsAg results was 30 (IQR 23–39). The proportion of positive HBsAg results increased with age (OR 1.02 (95% CI 1.02–1.03, *p* < 0.01)), as did the proportion of positive hepatitis C serology (OR 1.04 (95% 1.03–1.05, *p* < 0.01)).

The proportion of positive HBsAg results were significantly higher in males (601/10,586, 5.7, 95%CI 5.2–6.1) than females (40/1480, 2.7, 95% CI 1.9–3.7; *p* < 0.01), while the proportion of positive hepatitis C serology was not significantly different between males (499/10725, 4.7, 95% CI 4.2–5.1) and females 70/1428 (4.9, 95% CI 3.8–6.2, *p* = 0.675).

There were 10, 406 repeat HBsAg results over the 14-year period on 4923 individuals with a median time between the first and last test of 2.7 years (IQR 1.2–5.8). Of these, 7 (0.1, 95% CI 0.05–0.3) demonstrated HBsAg seroconversion (negative to positive serology), after a median time of 0.6 years (IQR 0.2–3.4 years). There were 10,578 repeat anti-HBc results performed over the 14-year period on 4830 individuals with a median time between first and last test of 2.7 years (IQR 1.2–5.6). Of these, 84 (1.7%, 1.4–2.1) demonstrated anti-HBc seroconversion (negative to positive serology), after a median time of 2.7 years (IQR 1.3–5.8 years).

There were 13,261 repeat hepatitis C tests performed over the 14-year period on 5606 individuals with a median time between first and last test of 3.0 years (IQR 1.5–6.1). Of these, 93 (1.7, 95% CI 1.3–2.0) demonstrated hepatitis C seroconversion (negative to positive serology), after a median time of 4.5 years (IQR 2.5–6.9 years).

There were 4921 individuals who had at least 2 complete hepatitis B serology tests. The median time between first and last serology was 2.8 years (IQR 1.3–5.8 years). Of those 1838 individuals who were non-immune on their first complete hepatitis B serology, 1237/1838 (67.3, 95% CI 65.1–69.4) remained non-immune on their last complete hepatitis B serology and 44/1838 (2.4, 95%CI 1.7–3.2) became anti-HBc positive.

There were 10,806 individuals who had both complete hepatitis B serology and hepatitis C serology. There were 21 cases (0.2, 95% CI 0.1–0.3) of coinfection.

## Discussion

We have shown that the proportion of positive serology over the 14-year period was 5.3% for HBsAg, and 4.7% for hepatitis C. This demonstrates the Northern Territory blood borne virus epidemiology within correctional facilities is somewhat different to national trends with a relatively higher proportion of positive HBsAg results, and a lower proportion of positive hepatitis C serology [2, 9]. We excluded 26 unique records which demonstrated hepatitis C antibody conversion from positive to negative. This represented 0.2% of the total records and could reflect either false positives or waning hepatitis C antibody and were not included in the proportion calculations.

There were 31,061 tests performed for HBsAb, but only 22,397 complete hepatitis B serology tests, consisting of HBsAg, anti-HBs, and anti-HBc. This has changed since 2007 whereby any request for any component of hepatitis B serology leads to the performance of the complete serology. This has allowed more complete interpretation of one’s serological status. This is important as complete serological assessment has been shown to be suboptimal in our region but is currently being addressed with data linkage and computerised coding [12].

The high proportion of positive HBsAg would be consistent with the high prevalence hepatitis B in the Northern Territory among Aboriginal and Torres Strait Islander people. Northern Territory wide prevalence of HBsAg is estimated to be 6.08% in Aboriginal and Torres Strait Islander people [13], and 84% of the prison population in the Northern Territory identifies as Aboriginal or Torres Strait Islander [11]. The higher proportion of positive HBsAg found in males compared to females is also consistent with overall Northern Territory and worldwide trends [13, 14].

The relative lower proportion of positive hepatitis C serology found in the Northern Territory correctional facilities compared to nationwide rates is interesting and is likely explained by lower rates of injecting drug use. The national prison entrants’ blood-borne virus survey showed that a lower proportion of prisoners reported a history of injecting drug use in the Northern Territory (7%) compared to other jurisdictions (> 40%) [2, 9]. Remote dwelling Aboriginal and Torres Strait Islander people also have lower risk of intravenous drug use than metropolitan counterparts [15, 16]. Despite this, surveillance is still required as there is a higher proportion of positive hepatitis C serology in Aboriginal and Torres Strait Islander people who inject drugs, compared to non-Indigenous counterparts, and injecting drug use is a strong risk factor for hepatitis C in prisons [3, 17]. The burden of hepatitis C is also increasing in Aboriginal and Torres Strait Islander people with age standardised rates of notification increasing 43% between 2011 and 2015 to 167 per 100, 000 population, compared to non-Indigenous people, which decreased 10% to 36 per 100,000 population [18]. Indeed, while the rate of injecting is low (3%) in Aboriginal and Torres Strait Islander people aged 16–29, it is associated with prison involvement and is higher than general population estimates [19].

Our results have shown there is opportunity to immunise individuals to hepatitis B while in correctional facilities in this region. Where there was at least two separate complete serology for hepatitis B over the study period (*n* = 4921), we found that of the 1838 who were non-immune on first result, 1237 remained non-immune on the last result during the study period. Additionally, in those non-immune on first testing, 44/1838 (2.4%) showed evidence of exposure (anti-HBc positive) on final serology. Immunisation to hepatitis B has been shown to be successful if given as an accelerated schedule in a correctional facility [20], while transmission of hepatitis B has been demonstrated in correctional facilities and has been associated with outbreaks in these settings [21, 22]. Further research and resources are required to assess the outcomes of immunisation for hepatitis B in the Northern Territory correctional facilities. This may require linkage mechanisms to remote communities to complete vaccine schedules if period of prison stay is short. The lower proportion of positive HBsAg among younger ages seen in our study is likely reflective of the success of universal vaccination in the Northern Territory, which has been in place since 1990 [23]. The reduced proportion of positive anti-HBc in younger people has also been demonstrated in prisons located in other Australian jurisdictions [24].

The proportion of records with positive serology for hepatitis B and C infection remain relatively high, yet hepatitis B and C serology has been performed less frequently since 2015. This decrease is likely related to a change in practice in which testing was taken a few days after reception, rather than on the day of reception, and was risk based. Universal offers of testing have been associated with increased diagnosis and treatment of hepatitis C in prison [25], while the national hepatitis B strategy aims to increase the proportion of people living with chronic hepatitis B who are diagnosed to 80% and list custodial settings as a priority [7]. For many patients, incarceration may present a rare opportunity to be tested and treated. We would therefore strongly support policy which increases testing rates in these facilities to maximise the opportunity to treat such populations, while also addressing issues surrounding risk behaviour, pre and post-test counselling and stigma [26]. The findings presented in this study have led to a change in testing policy in the Northern Territory in January 2020. Testing is now universal rather than risk based for both viruses, and prompts have been added to each electronic medical record to remind the provider to check previous hepatitis B results and perform if not complete.

The limitations of this study include its retrospective nature, and there was incomplete data in some years of the study period. This was due to testing being performed at another provider during those years and we did not have access to these results. There is a very remote chance that the conversion of the 2 × 2 name code and date of birth to a unique study number may have attributed two records to one individual when it was two different individuals. As duplicates were removed by each year and publicly available information was used to determine the number of prisoners entering each year, if a prisoner entered in the following year and were not tested as they had been tested the year prior and were positive, the proportion tested, and the proportion positive may have been affected. However, given the large number of tests and the proportion of positives over the entire 14-year period was not too dissimilar to the yearly proportions of positives, we suspect this was not a large effect. We did not have data on viral loads and can only make comments on the proportion of positive serological markers of infection rather than infection itself. Data on epidemiological characteristics from the records with positive serology were limited and we did not have data on the Aboriginal and Torres Strait Islander status of those tested. We were therefore not able to analyse risk factors for these markers of infection beyond age and gender.

## Conclusion

There is a relatively higher proportion of positive serological markers of hepatitis B infection, and a relatively lower proportion of positive hepatitis C serology in the Northern Territory’s correctional facilities compared to other Australian jurisdictions. As the proportion of prisoners tested for hepatitis B and C has decreased recently, and a high proportion of prisoners remain non-immune to hepatitis B, there is opportunity to increase testing and Hepatitis B vaccination rates in the prison population of the Northern Territory.

## Data Availability

The datasets are available from the corresponding author on reasonable request.

## Abbreviations

HBsAg: hepatitis B surface antigen
anti-HBc: hepatitis B core antibody
anti-HBs: hepatitis B surface antibody
